# Current pediatric rheumatology fellowship training in the United States: what fellows actually do

**DOI:** 10.1186/1546-0096-12-8

**Published:** 2014-02-10

**Authors:** Anjali Patwardhan, Michael Henrickson, Laura Laskosz, Sandy DuyenHong, Charles H Spencer

**Affiliations:** 1 Department of Child Health, University of Missouri Medical Center, Columbia, USA; 2 Cincinnati Children’s Hospital/University of Cincinnati, Cincinnati, USA; 3 Manager, Committee and Sections, American Academy of Pediatric, Washington, USA; 4 University of Iowa Children’s Hospital, Iowa City, IA, USA; 5 Nationwide Children’s Hospital/Ohio State University, Columbus, USA

## Abstract

**Background:**

Pediatric Rheumatology (PR) training in the US has existed since the 1970’s. In the early 1990’s, the training was formalized into a three year training program by the American College of Graduate Medical Education (ACGME) and American Board of Pediatrics (ABP). Programs have been evaluated every 5 years by the ACGME to remain credentialed and graduates had to pass a written exam to be certified. There has been no report yet that details not just what training fellows should receive in the 32 US PR training programs but what training the trainees are actually receiving.

**Methods:**

After a literature search, a survey was constructed by the authors, then reviewed and revised with the help members of the Executive Committee of the Rheumatology Section of the American Academy of Pediatrics (AAP) using the Delphi technique. IRB approval was obtained from the AAP and Nationwide Children’s Hospital. The list of fellows was obtained from the ABP and the survey sent out to 81 current fellows or fellows just having finished. One repeat e-mail was sent out.

**Results:**

Forty-seven fellows returned the survey by e-mail (58%) with the majority being 3rd year fellows or fellows who had completed their training. The demographics were as expected with females > males and Caucasians> > non-Caucasians. Training appeared quite appropriate in the number of ½ day continuity clinics per week (1–2, 71%), number of patients per clinic (4–5, 60%), inpatient exposure (2–4 inpatients per week, 40%; 5 or greater, 33%), and weekday/weekend call. Fellows attended more didactic activities than required, had ample time for research (54% 21-60/hours per week), and had multiple teaching opportunities. Seventy-seven percent of the trainees presented abstracts at national meetings, 41% had publication. Disease exposure was excellent and joint injection experience sufficient.

**Conclusions:**

Most US PR training programs as a whole provide an appropriate training by current ACGME, American College of Rheumatology (ACR), and ABP standards in: 1) number of continuity clinics; 2) sufficient on-call activities for weekday nights and weekends; 3) joint interdisciplinary conferences; 4) electives 5) didactic activities; 6) scholarly activities; and 7) exposure to diverse rheumatology diseases. Areas of concern were uniformity & standardization of training, need for a customized PR training curriculum, more mentorship, free electives, training in musculoskeletal ultrasound, need for a hands-on OSCE certification exam and more exposure to ACGME competencies.

## Background

Pediatric Rheumatology (PR) has existed as a subspecialty in the United States (US) since the 1960’s but was officially recognized as a separate subspecialty in the US in 1990. It has grown rapidly and yet remains one of the smaller pediatric subspecialties. The total number of trained pediatric rheumatologists (PRs) in the USA is less than 300 after being ~40 in 1976, and 90% of these are located in large cities [1]. There remains a major gap between demand for services of trained pediatric rheumatologists and the supply of these services in the US and worldwide [2-4]. This inadequate supply limits patient access to PR subspecialty care and places great pressure on the PR work force available [5,6]. In the US, a child with rheumatic disease must travel an average of 57 miles to be seen by a PR compared to an average of 25 miles for a child needing any other subspecialty [5].

In a three part series, Henrickson addressed policy challenges for the PR workforce in the US in 2012. He emphasized this limited access for patients in the US, the shortcomings of PR education for students, residents and community physicians, the problems facing expansion of the PR field, and how these shortages impact medical care of these PR patients [6-8]. Other policy research has focused on resident and medical school training in musculoskeletal disease and likely under-appreciation of the utility and importance of PR for education and patient care in US medical schools and medical centers [9-11].

The PR fellowship training positions have increased from 25 in 1997–1998, to 58 in 2004–2005, and to 75 in 2012. For 2011–12, there were 30 Accreditation Council for Graduate Medical Education (ACGME) accredited PR training programs in the US and each has 1–2 fellowship positions available yearly [12]. However, each year approximately one fourth of the fellowship positions may remain unfilled. This gap is due to a limited number of applicants in part due a lack of trainee awareness of these opportunities and/or a lack of consistent funding for these fellowship positions.

The American Board of Pediatrics (ABP) establishes the subspecialty training requirements for

PR Board Certification Examinations [13,14]. These ABP subspecialty certification examinations are taken during fellowship as in-service exams and after the fellowship as the certifying board exam. More examinations follow every 10 years as part of Maintenance of Certification. These exams assess mostly knowledge content and less practical clinical skills than the certification exams in the United Kingdom and other European countries [15]. The American College of Rheumatology (ACR) curriculum for rheumatology training is primarily oriented to adult care with a pediatric rheumatology supplement included [16]. Its mission statement is that all fellowship programs should train fellows that:

1. Are clinically competent in the field of rheumatology.

2. Are capable of working in a variety of settings.

3. Possess habits of life-long learning to build up on their knowledge, skills and professionalism. Based on the mission statement, the goals of the training were structured in these three areas. Finally objectives were developed in line with the goals.

The curriculum directs the achievement of these via the following recommended experiences:

A. The inpatient rheumatology experience.

B. The ambulatory rheumatology experience.

C. Ambulatory rotation with other clinical subspecialties.

D. Didactic conferences.

E. A research experience.

There are six core areas in the ACR guidelines including clinical (outpatients, inpatients, on call commitments), procedures (joint injections and musculoskeletal ultrasound), didactics (journal clubs, conferences, lectures, grand rounds and afternoon lectures), rotation with other clinical subspecialties, academics (scholarly activities, research, presentations in national and regional meetings and availability of mentorship, publications & involvement in teaching PR), and training and evaluations in generic/transferrable skills (such as communication, leadership, and ethics).

The US pediatric rheumatology fellowship training programs have used the ABP and ACR requirements to design each of their fellowship experiences. Until this year, US PR fellowship programs have been individually evaluated by the ACGME every 3–5 years using a detailed written description of each fellowship known as program information forms (PIF) plus an onsite visit. These reports on each program are available to individuals in the ACGME, hospital administrations, and perhaps some trainee candidates who ask for program information, but there has been no compilation available on how the US PR programs individually and as a group are meeting the PR ACGME requirements. Also, the exact information provided for the ACGME by each PR program in their PIF may vary to some degree from program to program. It was our belief that it would benefit the training of pediatric rheumatology fellows, and thereby the development of PR, if we took a survey snapshot of fellowship qualitative and quantitative achievements and outcome data. This data may also be useful as pediatric rheumatology programs adjust to the new ACGME evaluation standards of milestones and self-evaluation. In collaboration with the American Academy of Pediatrics (AAP) Section on Rheumatology, we conducted a nationwide observational, cross-sectional survey of PR fellows. The primary objective was to measure training specifics in terms of exposure and experience in six areas of training, including didactics, academics, clinical (outpatient and inpatient experience and on-call responsibilities), procedures, electives, and research. The secondary objectives were to obtain ethnic and demographic data. A future goal is to use this data to develop quality benchmarks and identify areas for improvement for the programs in general and for each program individually in the framework of the new ACGME accreditation standards. This data may also assist new fellowship programs in the US and worldwide in developing and improving their PR training programs.

## Methods

We first performed a literature review of other US pediatric subspecialty fellowship training publications to determine how best to obtain information on nationwide training variability. We developed survey questions to elicit objective responses as well as comments. We designed the survey and then used the Delphi technique among the authors and AAP Rheumatology Section Executive Committee members to improve the survey instrument. We obtained approval from institutional review board (IRB) of the AAP and Nationwide Children’s Hospital. We contacted eighty-one all current or just graduated PR fellows by emails in 2012 inviting their consent to participate in the anonymous online survey using Survey Monkey. We sent out the initial survey and then one reminder over the next four weeks to achieve a 58% response rate (47/81). One limitation of the survey was that due to an IRB request there was a limit to reminders we could send; only one reminder were sent to all the 81 fellows. Results were checked for accuracy both manually and using the survey software.

## Results

### Fellowship stage

Forty-six trainees responded to this question. Thirty-seven percent (18/46) had completed fellowship, 34% were 3rd year fellows (16/46), 5% (2/46) completed four years of training (likely medicine/pediatrics training), 16% (7/46) were 2nd year fellows and 7% (3/46) were 1st year fellows. We considered this sample a homogenous and comparable population of trainees from in view of the current training structure. There were not enough respondents from the first year of fellowship to use in the quantitative analysis. Rather we looked at their fellowship experience qualitatively, comparing their experience to the fellowship recommendations of the American Board of Pediatrics.

### Demographics

Table 1 lists the years after medical school of 40 fellows. The great majority of the respondents graduated from medical school 6–8 years prior to this survey.

**Table 1:** Years after graduation from medical school of rheumatology fellows surveyed

Years	4	5	6	7	8	9	10	11
# Fellows	2	2	6	14	8	3	2	3
% Fellows	5	5	15	35	20	7.5	5	7.5

Total respondents = 40.

### Gender

Forty-four trainees responded to this question. The female (33/44) to male (11/44) ratio of the respondents were 3:1.

### Race

Of 43 respondents 29 (68%) were Caucasian, 7 (16%) Asian-Indian, 4 (9%) Hispanic, 2 (4%) other, and one (2%) Asian-American. No respondent selected the African-American or Native American options.

### Attendance of fellows at half-day continuity rheumatology clinics

Of forty-six trainees who answered this question, 13 (28%) attended 1 half-day clinic per week, 20 (43%) attended 2 half-day clinics, 7 (15%) attended 3 half-day clinics, and 4 (8%) attended 4 half-day clinics, and 2 (4%) attended 6 half-day clinics per week. Thus the great majority of the fellows had 1–2 continuity half-day clinics per week.

### The mean number of patients seen per half-day clinic

Of the 46 respondents, most had 4 (14/46, 30%) or 5 (14/46, 30%) patients in the average half-day clinic. Eight respondents had 6 patients per clinic and eight only 3 patients. One respondent each averaged 7 or 8 patients per clinic.

### The average number of patients seen in an inpatient setting per week

The fellows saw a range of inpatients in a week in the inpatient services according to the responses of 45 trainees. Twelve (27%) would average 1 patient per week in the inpatient setting while 18 (40%) had 2–4 patients on the rheumatology service in a week. Fifteen (33%) were busier with 5 or over patients per week on the inpatient service.

### Didactics

The question attempted to capture the total number of hours per week that trainee spent in didactic activities, e.g. lectures, grand rounds, journal clubs. Of the 45 trainees who responded, six (13%) spend two hours in didactic activities, sixteen (36%) spent an average of 3 hours, thirteen (29%) spent 4 hours, and 10 (22%) spent 5 hours or more in these activities.

### Research

This question enquired about the total number of hours per week that each trainee usually spent on research activities such as literature research, data collection, clinical or bench research, or other such activities. Of the 44 respondents, 19 (43%) reported spending 20 hours or less per week in research activities, 19 (43%) reported 21–40 hours per week, 5 (11%) 41–60 hours per week, and 1 (2%) 60 hours or more each week.

### Combined clinical conferences

This question addressed with whom the trainees have had combined clinical or research conferences with other subspecialties. More than one answer was acceptable.

Of 42 trainees who answered this question, 32 (81%) trainees had joint conferences routinely with adult rheumatologists, 25 (60%) had conferences with radiology, 20 (48%) with nephrology and 18 (43%) with histopathology.

### Journal club presentations

Of the 45 trainees who responded, there was a wide range of journal club presentations each year with no particular trends and no relation to the year of fellowship. Most trainees (34/45 or 76%) presented at 2–4 journal clubs per year.

### PR topic lectures presentation

PR topic lectures presented by fellows involved multiple venues for 41/42 or 97% of the trainees who responded. The most common venue was resident teaching (39/45, 88%). Medical student teaching conferences were next (26/45, 59%), followed by adult rheumatology/internal medicine grand rounds (22/45, 50%), pediatric grand rounds (8/45, 19%), and others (7/45, 16%) that included pediatric noon conferences and Arthritis Foundation sponsored conferences for primary care physicians.

### Abstract Presentation at a national/regional scientific meeting

Thirty-four of 43 fellow respondents (77%) had a chance to present an abstract at a regional or national scientific meeting. The range of numbers of abstracts presented during their fellowship varied from 11 (32%) trainees presenting one abstract to 2 (6%) presenting 8 abstracts in their fellowship. The median number of abstracts presented by fellows was 2 abstracts.

### Publications during fellowship

Of the 44 trainee respondents, 18 (41%) published an article during their fellowship. Only 18 trainees responded to the follow-up question on the number of articles published. Eleven trainees published one manuscript (61%) with one trainee publishing five (6%) with the remaining trainees publishing 2–4 manuscript (33%). The median was one publication.

### Availability of mentorship resource

Of the forty-seven trainees who answered this question, thirty-six (78%) trainees reported that they had no access to mentors other than their scholarship oversight committee and fellowship program director. Six (12%) fellow trainees had mentors outside their PR subspecialty and 5 (11%) had their research mentors as their mentors.

### Procedures: the average number of joint injections performed per month

Of the 45 fellow trainees who responded, 25 (56%) reported doing one joint injection/month (56%), 17 (37%) reported doing two to four joint injections/month, and 3 trainees reported doing five or more joint injections/month (6%). Table 2 describes the joints injected most often by trainees. The knees and ankles were the predominant joints injected. The fellows had less chance to inject the hip, shoulder, finger, and TMJ joints (Table 3).

**Table 2:** Extent of exposure of more common pediatric rheumatic diseases during entire fellowship

**Diseases/Syndromes (Column A)**		**Number of patients with the corresponding disease (Column B)**			
		**0–5 patients**	**6–10 patients**	**11–20 patients**	**>20 patients**
Systemic JIA	Percentage of fellows who saw the patients in each group of column -B with diseases listed in column –A	16%	47%	28%	9%
Poly JIA		4%	7%	14%	75%
Oligo JIA		4%	0%	15%	81%
Enthesitis-related JIA		7%	16%	25%	52%
SLE-New case		30%	34%	32%	4%
SLE-existing diagnosis		11%	14%	32%	43%
SLE with nephritis		21%	25%	27%	27%
JDM-new case		57%	30%	4%	9%
JDM-existing diagnosis		31%	30%	25%	14%
Scleroderma		47%	29%	20%	4%
Streptococcal syndromes		20%	39%	37%	4%
Resistant HSP		54%	30%	9%	7%
Kawasaki Disease		21%	46%	19%	14%
Sarcoidosis		61%	34%	5%	0%
Pain syndromes		2%	16%	18%	64%

(44 responded).

*Abbreviations*: *JIA* juvenile idiopathic arthritis, *SLE* systemic lupus erythematosus, *JDM* juvenile dermatomyositis, Streptococcal syndromes include rheumatic fever, post-streptococcal reactive arthritis and PANDA (Pediatric Autoimmune Neuropsychiatric Disorders Associated with Streptococcal infections), *HSP* Henoch Schӧenlein purpura.

**Table 3:** Extent of exposure to joint injections during fellowship years of each fellow

**Joints**	**Respondents who injected 0–3 joints (%)**	**% injected 4–6 joints**	**% injected 7–12 joints**	**% injected >12 joints**
TMJ	100	0	0	0
Shoulders	92	6	0	2
Elbows	71	26	3	0
Wrists	52	34	7	7
MCPs	82	5	5	8
PIPs	74	11	11	4
IP’s	77	18	2	3
Hips	100	0	0	0
Knees	7	7	26	60
Ankles	44	37	7	12

Total respondents = 44.

Note: The experience is quantitated by dividing the experience in different subgroups based on “Number Joints injected”. The numbers in italics in each subgroup denote% of responders in each subgroup.

### Formal training in musculoskeletal ultrasound

Nine of the 46 trainee respondents (20%) completed formal training in musculoskeletal ultrasound during the fellowship including one fellow who paid for this training her/himself.

### Weekday and weekend on call service per month

The average number of weeknight pager calls from home per month varied widely. Of 44 respondents, seven fellows (16%) were on pager call 3–6 nights per month, twelve (27%) did 7–9 nights per month, 6 (14%) did 10–14 nights per month and 3 (7%) did 15 or more on call nights per month. One trainee did no week-night on call during her/his fellowship.

Trainee exposure to weekend pager on call per month also had a wide range as well. Saturdays and Sundays were considered two separate weekend days. Sixteen fellow trainees (36%) did one weekend day per month, 17 (38%) did 2 weekend days on call per month, 4 (9%) did 3 weekend days and 4 trainees (9%) did 4 weekend days on call per month. Forty-three trainees had to do inpatient rounds during their weekends on call with their attending rheumatologists and apparently, if the question was answered correctly, one trainee did not.

### Participation in electives

O the 44 trainees who answered this question, thirty-two trainees (72%) indicated that their program allowed them to take electives while 16 (28%) trainees responded that they did not have the option of taking electives. These electives were 2 to 12 week blocks of training time that the trainee could have an educational experience outside of pediatric rheumatology either of their own choice or a required elective. Of the 32 fellow trainees who took electives, 26 took required electives which were built into their training program and were not selected by personal preference.

Adult rheumatology was the most common required elective (11/32, 43%) followed by immunology (4/26, 15%), radiology (3/26, 12%), nephrology (3/26, 12%), sports medicine (2/26, 8%), histopathology (1/26, 4%), pain medicine (1/26, 4%), and orthopedics (1/26, 4%). Twenty of the trainees taking required electives had 1–2 week electives, 5 had 3–4 weeks electives and 1 had a 12 weeks elective.

When free to choose an elective, these were the disciplines noted in a comment section of the survey as electives fellows have taken or might want to take: immunology, sports medicine, pain centers, ultrasound, nephrology, physical therapy, adult rheumatology, histopathology, administrative, orthopedics, vasculitis, and pediatric rheumatology in another institution.

### Disease exposure

This question was in a tabular form and respondents were asked to fill in the average number of patients they cared for of each disease category during their fellowship using their recollection. We believe that this method is accurate as the trainees are required to keep a log of both their disease exposure on the inpatient service and in all clinics they attend [e.g., systemic lupus erythematosus (SLE), polyarticular juvenile idiopathic arthritis (poly JIA), and dermatomyositis (JDM)] as well as of those specific patients that they follow in their continuity clinic. These experiences for budding pediatric rheumatologists are often indelible. The data in Table 2 and Table 4 on the extent of disease exposure during fellowship years represent the cumulative analysis of all the individual responses. The experience is quantitated by dividing the experience in different subgroups based on “Number of patients seen”. The numbers in italics in each subgroup denote% of responders in each subgroup. These responses denote fellow’s total inpatient and outpatient contact with these diseases. The responses do not necessarily suggest that each fellow had continuously followed these patients in their own continuity clinics.

**Table 4:** Extent of disease exposure of less common pediatric rheumatic diseases and musculoskeletal syndromes during entire fellowship

**Diseases/Syndromes (Column A)**		**Number of patients with the corresponding disease (Column B)**			
		**0-5 patients**	**6-10 patients**	**11-20 patients**	**>20 patients**
Sjogren’s syndrome	Percentage of fellows who saw the patients in each group of column -B with diseases listed in column –A	72%	23%	5%	0%
New WG (GPA)		93%	7%	0%	0%
Existing WG Diagnosis		73%	22%	5%	0%
PAN		98%	2%	0%	0%
MPA		93%	7%	0%	0%
CNS vasculitis		82%	18%	0%	0%
Behcet’s syndrome		84%	14%	2%	0%
Churg-Strauss syndrome		100%	0%	0%	0%
Other ANCA-related syndromes		84%	8%	8%	0%
APLS		69%	21%	8%	2%
Periodic Fever syndromes		85%	14%	1%	0%
CRMO		72%	26%	2%	0%
MAS		91%	7%	2%	0%

(44 responded).

*Abbreviations*: *WG* Wegener’s Granulomatosis (GPA granulomatosis with polyangiitis), *PAN* polyarteritis nodosa, *MPA* microscopic polyangiitis, *CNS* central nervous system, *ANCA* anti-neutrophilic cytoplasmic antibody, *APLS* antiphospholipid syndrome, *CRMO* chronic recurrent multicentric osteomyelitis, *MAS* macrophage activation syndrome.

## Discussion

We know that there is wide variation in clinical practice among pediatric rheumatologists across Northern America and Canada [17-20]. This variation in practice may in part be due to fellowship training variability during the past 20 years. This variation is not necessarily bad and may be due to the programmatic strengths and weaknesses of each program. For example, several rheumatic diseases such as juvenile dermatomyositis, scleroderma, and systemic vasculitis illnesses are very unusual in children and adolescents. This may limit trainees’ exposure and consensus in practice. The developing pragmatic protocols in pediatric rheumatology, such as the Clinical Treatment Plans of the Childhood Arthritis and Rheumatology Research Alliance (CARRA), and similar protocols in Europe in the Pediatric Rheumatology International Trials organization (PRINTO) and Pediatric Rheumatology European Society (PReS), may help identify best practices. Consensus guidelines are just now being developed.

To answer this question of what fellows in the US actually do, this nationwide survey was conducted. Based on the ACR curriculum for rheumatology fellowship training in USA and Canada and the ABP rheumatology curriculum requirements, our survey was structured in a way that it collected information on all of these key areas. The open-ended questions with free text spaces were used to elucidate responses that would reflect the training experience in the six core areas. Participation in the survey (47/81-58%) was lower than hoped but may have been hampered by a low response rate from 1st year fellows. We believe that this low 1st year fellow response rate may have been due to their perception that they had a limited fellowship experience to share. For this reason, many 1st year fellows may have not elected to respond to the survey.

We found that the US fellowships as a group accomplish many requisite goals. We will focus on several survey results.

1) Ambulatory and inpatient experience

The recommendations on ambulatory experience are that fellows must conduct continuity clinics equivalent to a full day for the first twelve months of fellowship with an attending preceptor. Thereafter, they should maintain one half-day clinic for the rest of the two-year period in a supervised environment with their preceptor. Our survey results showed that 95.8% of the first year trainees have two or more (45/47) one half day clinics/week and met the requirements while only 2/47 had only a single half day clinic/week day and did not meet the requirement. Survey respondents noted that there was a chronic tension created by balancing outpatient clinic volume and educational activities, including case review time with the preceptor.

In our experience, there is a frequent standard of one hour for a new visit and 30 minutes for a follow-up visit. With these lengthy time constraints, fellows may suffer a substantial loss of learning time while patients were unavailable due to check-in and administrative functions [18]. The number of patients seen per half day clinic ranged from 3 (17%) to 8 (2%); the most common pattern was 4–5 patients/half day clinic (30%). Generally, half-day clinic durations range from approximately 3–6 hours in duration. Based on one hour for a new patient and 30 minutes for a follow-up visit, the optimal numbers of patients should be no more than 4-6/ half day clinic. Anything below this number may be an under-exposure and over this number may impede the learning process.

Inpatient experience appears quite adequate for our field of PR. Forty percent of the respondents reported 2–4 inpatients per week with 33% reporting 5 patients per week or more. We know from experience that these patients are often complex with high morbidity and this number of inpatients, though not high compared to other subspecialties, appears sufficient to provide the needed training.

2) Interaction with other disciplines

Regarding interdisciplinary interactions, the ACR specifically recommends shared and interactive experience with dermatology, orthopedics, rehabilitative medicine, and ophthalmology as these subspecialty services are frequently required in the care patients with rheumatic diseases. There are no specific recommendations regarding how much time the trainee should spend in these activities and whether these experiences should be in the form of joint conferences or elective experiences. The ACR also recommends joint conferences with a several subspecialties.

The survey results showed that all of the trainees had in-depth and varied experiences with joint conferences. Interestingly, the most common joint conferences were with adult rheumatologists, and pediatric subspecialties such as radiology, nephrology and with histopathology subspecialties and not with other disciplines such as dermatology, orthopedic surgery, physical and rehabilitation medicine, or ophthalmology as recommended by the ACR curriculum. the trainees indicated that they also would have also liked to have elective rotations in either immunology, sports medicine, ultrasound, physical therapy, medical administration, and orthopedic surgery, vasculitis, pain medicine, or pediatric rheumatology in another institution. Some trainees clearly would have preferred to expand their training beyond their current curriculum.

3) Electives

The experience with electives was not as positive as with joint conferences. Twenty-eight percent of respondents felt they did not have an opportunity to take electives. Also 40% of the respondents did not take any electives. For the respondents who did take electives, the choice was not usually based on their preferences or perceived needs. Rather, the electives were often built into the program. More flexibility in elective rotations may be appropriate.

4) Didactic activities

Didactic activities complement clinical work and constitute an essential aspect of the curriculum. Grand round presentations, journal clubs, lectures from other specialties (radiology, nephrology, pain management, dermatology, sports medicine, etc.) as well as case discussion sessions may promote discussion and new research ideas, reduce the knowledge gaps, and afford time for questions. The ACR recommends “at a minimum there should be at least one clinical, one basic science, one literature review (journal club) and one research conference each month”. Further, the ACR recommends that there should be speakers and participation from other specialties that rheumatology frequently interacts with including pathology and radiology. The Fellows are required to attend at least 60% of these conferences throughout the year.

Our survey results revealed that the fellow respondents impressively exceeded this recommendation of didactic activities. The average time in such activities ranged from 2 hours/week to 5 or more hours/week, or 8 hours/month to 20 hours/month, compared to the recommended 2.6 hours to 4 hours/month. Notably, the survey did not specifically ask if the ACR-recommended clinical, basic science, literature review (journal club) and research conference content goals were met. All the trainees presented in journal club (1 to 7 journal clubs/year) and most of the trainees (77%) presented in 2–4 journal clubs per year.

5) On-call experience

The ACR/American Board of Pediatrics (ABP) curriculum requires on-call experience without quantifying the requirement. Several factors affect this element of fellowship training including number of available clinical fellows, institutional and division policy, and the option of alternating month-long intervals on-call [13,16,21]. This survey did not discriminate amongst these options, instead seeking an aggregate number for each respondent. Overall, the on-call experience appeared quite adequate to train fellows to take night-time phone calls and weekend call, with only a few outliers. When a training program’s primary institution does not provide sufficient on-call experience, fellows may benefit from rotating to external sites to supplement this training requirement, though the logistics may be challenging. Rotation in different programs may also improve exposure to different rheumatic diseases common in certain specific cities and regions as well as different practice models [13,16,21].

6) Disease exposure

US PR fellows appear to have a sufficiently diverse exposure to many different disease categories (Table 2). The exposure for most programs appeared to be very appropriate for common PR diagnoses such as JIA, SLE, JDM, and pain syndromes. As expected, there was limited exposure to rare diseases in childhood such as scleroderma and systemic vasculitis other than Kawasaki disease or Henoch-Schonlein purpura. Overall, this is an encouraging result.

7) Scholarly activities

The ABP requires fellows to participate in scholarly activity. This is particularly critical in PR as the great majority of fellows work as faculty in medical schools after they finish fellowship, in contrast to adult rheumatology fellows. The core research curriculum should include a review of biostatistics process, ethics, methodology and application. Literature review, grant-writing, funding sources and the application process are also important components [18]. The ACR curriculum suggests that research-related activities should comprise 5-10% of the total time in the first fellowship year, and 75-80% during the fellowship years two and three. This time span should allow development of a scholarly research project. The ABP requires a scholarly oversight committee (SOC) that supervises and mentors any project. To meet the curriculum objective, the fellow must generate at least one “work-product” such as the peer reviewed publication in which the trainee played a substantial role, a dissertation thesis for an advanced degree, or an extra-mural grant application [16,22]. Regarding research experiences, the ABP recommends that fellows should have definite protected time for research to learn and develop a research hypothesis, methodology, and statistical analysis. During this phase of training, each fellow is expected to have the guidance of a research mentor.

In the ACR curriculum for fellows, there are no quantitative or qualitative specifications on how many hours per week each fellow should spend in research if the fellow wants to pursue a more research-focused career than if he/she wants to pursue a more clinical-focused career. Not surprisingly, the survey data demonstrated considerable variation in fellow research training in the US. The average number of hours per week appeared appropriate and sufficient with the time the trainees spent on research activities ranging from 20 hours (43%), to over 60 hours per week (2%) in the second and third year of fellowship compared to 1–2 hours per week in first year of fellowship. The number of responders from first year fellows was small (3/47).

While it is commendable that 77% of the fellows presented one abstract at a national meeting with some presenting multiple abstracts, only 41% published a single article during fellowship. We believe this output could be improved, though it is possible that some of the remaining 59% may publish an article on fellowship research post-fellowship.

8) Musculoskeletal ultrasonography

Training in musculoskeletal ultrasonography (MUS) may offer an opportunity to be on the cutting edge of rheumatology. There are a variety of MUS training programs, including at an ACR meeting, which can facilitate certification. As is apparent from this survey, few PR fellowship programs currently offer MUS training opportunities as only 20% of the respondents had such training.

9) Training in the six competencies

As an additional dimension of quality improvement, the ACR curriculum recommends training and proficiency in providing excellent healthcare service delivery. These are reflected in the six competencies now used to measure physician performance and skills in the US including patient care, medical knowledge, practice-based improvement, interpersonal skills and communication, professionalism, and system-based practice. Our survey indicates most programs do not currently provide dedicated time and training for these skills [15]. If a PR fellow trained in the US for residency, these areas are often covered exhaustively. Yet some PR fellows may not have done their residency training in the US and require additional training in these areas. There will be training needed in the new milestones and entrustable professional activities (EPA) as well.

10) Widely variable PR training

US PR fellowship training may improve by narrowing the spectrum of fellowship training experiences while not applying an overly-standardized experience or discouraging experimentation.

While the survey definitely shows that many trainees are getting excellent training, there are always improvements to be made. Several improvements might include:

1. Establish a PR fellowship curriculum-a formal curriculum may be needed that fits with the new ACGME guidelines with milestones and entrustable professional activities as well as maintenance of certification activities for the ABP.

2. Standardize training content-this will lead to less variation from region to region and program to program.

3. Establish training minimum standards-each program may vary widely in practice models, disease exposure, and availability of resources and funding for education for training fellows. But there should be minimum standards for the PR training experience, e.g., joint injections, on-call experience, disease exposure, and other important areas.

4. Increase hands-on training exams-more OSCE testing for certification should be considered for in-service and post-fellowship ABP exams.

5. Increase joint injection experiences.

6. Offer musculoskeletal ultrasound training-Each program should consider making ultrasound training mandatory and pay the requisite fees.

7. Promote mentorship beyond the program director-each program should identify a mentor for each fellow within the program and outside the program. The current program mentoring program of the ACR (AMIGO) may fulfill this need in the US for long-term outside mentorship.

8. Increase exposure to and training in the six ACGME competencies.

## Conclusions

Pediatric Rheumatology is a relatively young subspecialty and PR fellowship training has multiple areas that could be optimized. Recognizing what programs do well and what areas need improvement is the first step towards improving training. We surveyed PR fellows across the US to measure training specifics in terms of exposure and experience in six areas of training including didactics, academics, clinical (outpatient, inpatient and on-call experiences), procedures, electives, and research. We reviewed the ethnic and demographic details of the upcoming workforce. The programs did very well in fulfilling the recommendations of the ACR, ABP, and ACGME. As we anticipated, there was wide variation in training resources and the PR fellowship training. We recognize that this variation may be partly due to the absence of a universally accepted PR fellowship curriculum and objective measures for training and evaluation leading to poor universal standardization. These deficits may soon be addressed by the ongoing development of ACGME milestones and ongoing, redefined ABP certification efforts. It would be useful to develop a PR fellowship curriculum.

Each fellowship program may want to evaluate its own program compared to the data in this study. Each program and its fellows may find that changes may improve their training by interventions such as more joint injection procedures, improved research and general mentoring, increased on-call exposure, increased clinic time for the fellows, and ultrasound training. All PR training programs may be able to cultivate a system which can re-evaluate the PR fellowship training gaps across the country on an ongoing basis and lead to continuous quality improvement that will enhance our training programs. This survey may also help new PR fellowship training programs in other countries devise their training programs and improve their programs appropriately over time.

## Competing interests

We declare that there were no conflicts.

## Authors’ contributions

P conceived the project and designed the survey with multiple revisions with help from all co-authors. Survey results was sent out by L and results sent to P. Each author reviewed the results and discussed the results with P. P and S wrote and revised the paper with editing from H. All authors approved the final draft.
